# Time-course adaptive changes in hippocampal transcriptome and synaptic function induced by simulated microgravity associated with cognition

**DOI:** 10.3389/fncel.2023.1275771

**Published:** 2023-10-05

**Authors:** Rong Liang, Ling Wang, Qing Yang, Qing Xu, Shufan Sun, Haichen Zhou, Meiling Zhao, Jing Gao, Chenguang Zheng, Jiajia Yang, Dong Ming

**Affiliations:** ^1^Academy of Medical Engineering and Translational Medicine, Tianjin University, Tianjin, China; ^2^Tianjin Key Laboratory of Brain Science and Neuroengineering, Tianjin, China; ^3^Haihe Laboratory of Brain-Computer Interaction and Human-Machine Integration, Tianjin, China

**Keywords:** simulated microgravity, cognition, synaptic plasticity, RNA sequencing, hippocampus

## Abstract

**Introduction:**

The investigation of cognitive function in microgravity, both short-term and long-term, remains largely descriptive. And the underlying mechanisms of the changes over time remain unclear.

**Methods:**

Behavioral tests, electrophysiological recording, and RNA sequencing were used to observe differences in behavior, synaptic plasticity, and gene expression.

**Results:**

Initially, we measured the performance of spatial cognition exposed to long-term simulated microgravity (SM). Both working memory and advanced cognitive abilities were enhanced. Somewhat surprisingly, the synaptic plasticity of the hippocampal CA3-CA1 synapse was impaired. To gain insight into the mechanism of changing regularity over time, transcriptome sequencing in the hippocampus was performed. The analysis identified 20 differentially expressed genes (DEGs) in the hippocampus after short-term modeling, 19 of which were up-regulated. Gene Ontology (GO) analysis showed that these up-regulated genes were mainly enriched in synaptic-related processes, such as Stxbp5l and Epha6. This might be related to the enhancement of working memory performance under short-term SM exposure. Under exposure to long-term SM, 7 DEGs were identified in the hippocampus, all of which were up-regulated and related to oxidative stress and metabolism, such as Depp1 and Lrg1. Compensatory effects occurred with increased modeling time.

**Discussion:**

To sum up, our current research indicates that the cognitive function under SM exposure is consistently maintained or potentially even being enhanced over both short and long durations. The underlying mechanisms are intricate and potentially linked to the differential expression of hippocampal-associated genes and alterations in synaptic function, with these effects being time-dependent. The present study will lay the experimental and theoretical foundation of the multi-level mechanism of cognitive function under space flight.

## Introduction

Whilst in spaceflight, astronauts are subjected to a variety of environmental stressors, including microgravity, radiation, and frequent day-night shifts (Mader et al., 2011; Sihver et al., 2015). Microgravity is a key factor inducing changes in various vital brain functions. Numerous studies have investigated the cognitive function under the simulated microgravity (SM) environment, but no final consensus has been reached. Divergent findings have reported that the effect of microgravity on cognitive function is deteriorating or improving (Wang et al., 2015). Some study suggested that the neurocognitive abilities of subjects were enhanced during short parabolic flights (Wollseiffen et al., 2016), and our previous results also showed that long-term SM mice exhibited enhanced spatial cognitive capabilities (Liang et al., 2022). Conversely, other investigations reported no impact on cognitive performance from 8 days of −6° head low bed rest (Manzey and Lorenz, 1998). Furthermore, additional animal studies revealed that the application of SM to rodents can potentially lead to cognitive dysfunction (Qiong et al., 2016; Zhang et al., 2018b; Kiffer et al., 2020). These contrasting findings prompt the question as to whether the changes in cognitive function under SM are contingent on time duration and condition variation, which previous studies have not investigated.

Based on this, numerous studies have investigated the possible mechanisms behind alterations in the hippocampus, a crucial brain region involved in cognitive function, under different SM exposure time. For example, a comprehensive proteomic analysis revealed that 7 days of SM exposure resulted in changes in hippocampal structural proteins in mice accompanied by a loss of proteins associated with cell metabolism (Sarkar et al., 2006). SM for 14 days caused changes in hippocampal synapse-related genes and impaired cytomorphological characteristics of CA1 (Frigeri et al., 2008; Ranjan et al., 2014). After 21-day SM, it induced changes in neurotransmitters (GABA and Glu) and metabolism-related proteins (Wang et al., 2015, 2016, 2017b). However, the current studies only examined alterations in gene-to-protein expression at predetermined time intervals of SM exposure and could not ascertain the precise overarching principle along with modeling time. The specific molecular mechanism underlying temporal changes is not unclear.

Thus, the primary aim of this study was to explore the changes in cognitive performance, specifically working memory and advanced spatial learning, under short- and long-term exposure to SM. Subsequently, to reveal the changing regularity over time and unravel its potential mechanism of action, the synaptic plasticity of the hippocampal CA3-CA1 synapse was evaluated, and combining the model with genome-wide RNA sequencing to determine the changes in hippocampal synaptic function and gene expression. Our study not only provides crucial insights into hippocampal neural mechanisms that contribute to cognitive shifts during microgravity exposure, but also facilitates the development of effective countermeasures for future long-term space stays.

## Materials and methods

### Animals

All animals were purchased from the Beijing Vital River Laboratory Animal Technology Co., Ltd. Six- to eight-week-old C57BL/6J male mice and Wistar rats were group-housed four to five in a cage under normal light-dark (23°C, 12-h light/12-h dark cycle) with *ad libitum* food and water. All animal experiments comply with the ARRIVE guidelines and are carried out in accordance with the U.K. Animals (Scientific Procedures) Act, 1986 and associated guidelines, EU Directive 2010/63/EU for animal experiments, or the National Research Council’s Guide for the Care and Use of Laboratory Animals. Thirty-four mice and 30 rats were used in the main experiments of this study. All efforts were made to minimize the number of animals and their suffering.

### Simulated microgravity exposure

To test the change with time, the SM animal model was respectively built for 2, 3, or 4 weeks. The animal’s tail was suspended by 30° to simulate the physiological effects of weightlessness. The method was similar to previous studies (Sandal, 2001; Yang et al., 2021; Liang et al., 2022, 2023). In simple terms, each animal was fixed with a mousetrap, in which only the tail was exposed. Wash the tail with warm water, and wipe it with a paper towel. The animal’s tail was then wrapped in breathable gauze, one end of the nylon rope was secured with medical tape, and the other end was attached to the beam of the tail-hanging cage. The keychain allowed the animal to freely rotate 360° in the horizontal direction, and by adjusting the length of the nylon rope, the animal’s torso was at a 30° angle to the ground, thus achieving the effect of weightlessness (Liang et al., 2022). In this case, exposure to SM for 2 weeks was considered as short-term SM exposure, while exposure for 3–4 weeks was long-term SM exposure.

### Y-maze test

The Y-maze test was used to measure working memory performance in animals (Morgan et al., 2018).

#### Novel arm test

The test is based on the rodent’s instinctive interest to explore unexplored areas. Animals were positioned in one arm of the maze (start arm) and allowed to explore the maze with one closed arm (novel arm) and one open arm (familiar arm) for 5 min (training stage). After 2 h of the training stage, the novel arm was opened, and the animal was reintroduced to the maze and allowed to explore all three arms of the maze freely for 5 min again (test stage). During the test stage, the percentage of distance traveled in the novel arm was analyzed.

#### Spontaneous alternation test

The animal was allowed to begin the test facing the wall of one arm, and the animal movements in the maze were recorded by a camera for 5 min. The actual interactive behavior was defined as the animal entering three consecutive arms in sequence, such as ABC, BAC, or CBA, but not ABA, BCC, or CBC. The alternate accuracy = [(number of actual alternations) / (number of total arm entries − 2)] × 100%, as an indicator to evaluate working memory performance (Zhang et al., 2018a).

After each session, the apparatus was wiped with 75% alcohol and water to avoid interfering with the next experiment.

### Morris water maze test

The Morris water maze (MWM) test is performed to detect hippocampus-dependent spatial cognition in SMmodel rats. It was performed as described previously (Yang et al., 2019). The experimental equipment is provided by Beijing Zhongshi Dichuang Technology Development Co., Ltd. The whole task consisted of two consecutive stages: initial training (IT) and space exploring test (SET). In the IT stage, escape latency (the time required to find the platform) and the swimming speed were recorded. In the SET stage, quadrant dwell time (the percentage of time spent in the target quadrant) and platform crossings (the number passing platform area) were measured.

### Open field test

The open field is a black box (90 × 90 cm), and for data analysis, the total area is equally divided into 16 equal squares virtually, a central area (45 × 45 cm), and a peripheral area (22.5 cm on each side). Each animal was gently placed in the center of the field, and its route was recorded for 5 min. Central area visits in the central zone were measured (Wang et al., 2020).

### Elevated plus maze test

In the test, animal was placed in a standard elevated plus maze (EPM) sized maze, respectively. Animal was allowed to explore the maze freely for 5 min, and the percentage of entries into the open arms was assessed.

### *In vivo* electrophysiological recording and analysis

The day after the behavioral experiment, electrophysiological tests of the hippocampus were performed. The recording and stimulating electrodes were implanted in the hippocampal CA1 (AP: −4.2 mm, ML: +3.5 mm, DV: −2.5∼3.0 mm) and CA3 (AP: −3.5 mm, ML: +2.5 mm, DV: −2.0∼2.5 mm) regions. Local field potential (LFP) signals were sampled simultaneously in both CA1 and CA3 at a 1-kHz sample frequency. Before the LTP induction, the test stimuli was delivered to the CA1 region every half a minute to evoke a response (range 0.05∼0.5 mA). Then the stimulus density, which can evoke a response of 50% of its maximum amplitude, was delivered at single-pulse stimulation to record a 15-min baseline (one stimulating pulse every 30 s). Afterward, theta-burst stimulation (TBS) consisting of 30 bursts at 5 Hz was performed to induce LTP, and each burst contained 12 pulses at 200 Hz. The electrophysiological data were measured in DataWave SciWorks (A-M Systems, USA). All the LFP data processing was conducted offline using custom routines in R2014a MATLAB (MathWorks). The power spectral analysis, phase-locking value (PLV), and coherence of theta and gamma rhythms were analyzed. The details of the computing methods were described in the previous study (Wang et al., 2021).

### RNA sequencing and analysis

After perfusing with PBS solution for 8 min, the mice were decapitated and the whole hippocampus (HPC) was taken out. Immediately after dissection, the tissue was frozen at −80°C for subsequent transcriptome sequencing. The HPC from two mice of each group was pooled to make one RNA sample and a total three pairs of RNA samples (three normal mice, three SM mice, thus total six mice per group) were processed for RNA sequencing. RNA extraction, library preparation, cluster generation, and sequencing were performed by Novogene (Beijing, China).

Raw data (raw reads) of fastq format were firstly processed through fastp software. In this step, clean data (clean reads) were obtained by removing reads containing adapter, reads containing ploy-N and low quality reads from raw data. At the same time, Q20, Q30, and GC content the clean data were calculated. All the downstream analyses were based on clean data with high quality. Differential expression analysis of two conditions/groups (two biological replicates per condition) was performed using the DESeq2 R package (1.20.0). DESeq2 provided statistical routines for determining differential expression in digital gene expression data using a model based on the negative binomial distribution. The resulting *p*-values were adjusted using Benjamini and Hochberg’s approach for controlling the false discovery rate. Genes with an adjusted *p*-value ≤ 0.05 and | log2 fold change| ≥0.5 found by DESeq2 were assigned as differentially expressed (Xiao et al., 2020).

Gene Ontology (GO) enrichment analysis of differentially expressed genes (DEGs) was implemented by the clusterProfiler R package, in which gene length bias was corrected. GO terms with corrected *p*-value less than 0.05 were considered significantly enriched by DEGs. The Kyoto Encyclopedia of Genes and Genomes (KEGG) is a database resource for understanding high-level functions and utilities of the biological system, such as the cell, the organism and the ecosystem, from molecular-level information. We used the clusterProfiler R package to test the statistical enrichment of differential expression genes in KEGG pathways (He et al., 2020).

### Quantitative real-time PCR

To verify transcriptome sequencing data, three DEGs in each of the hippocampal brain regions of short- and long-term SM were selected for validation by quantitative real-time PCR (RT-qPCR), as previously mentioned (Li et al., 2020; Ding et al., 2021). Specific primers were designed according to reference sequences in the NCBI database with Primer Bank and are listed in Table 1. The primers were synthesized by Sangon Biotech (China). Total RNA was extracted from the HPC using TRIzol reagent and reverse-transcribed into cDNA using Thermo Scientific RevertAid First Strand cDNA Synthesis Kit (#K1622, Thermo, USA). After reverse transcription, RT-qPCR was carried out on Applied Biosystem Real-Time PCR System (CFX96, Bio-Rad, USA). The 15 μl reaction system contained 7.5 μl of 2x ChamQ Universal SYBR qPCR Master Mix (#Q711, Vazyme, China), 0.3 μl of each primer (forward and reverse), 5 μl of cDNA template, and 1.9 μl of nuclease-free deionized water. The three-step method was performed for amplification with denaturation at 95°C for 30 s, followed by 40 cycles at 95°C for 10 s and 60°C for 30 s; and final elongation at 95°C for 15 s, 60°C for 1 min, and 95°C for 15 s. The relative mRNA expression levels of selected genes were calculated with the 2^–ΔΔCq^ method using GAPDH as an internal reference. Each sample was tested in triplicate.

**TABLE 1:** Primer sequences used for RT-qPCR experiments.

Items	Direction	Sequences
StxbpSI	Forward primer	TCTGCTGTGCTGTCCAAGTAAGG
	Reverse primer	ATGTGCCCACTCCCTCTCCTC
Epha6	Forward primer	TCAAGCAGGTCAGAAAGCAGGAG
	Reverse primer	GAGCAAGCCAGGGAAAGCAAAC
Kcnj6	Forward primer	TGAGGAGAGGCAGGTGGTAAGG
	Reverse primer	AGGTCAAGGCACATACAGCAAGG
Deppi	Forward primer	AGAGGAAGTAGGAGGGTGTCAGG
	Reverse primer	TTTCCCGAATCGTTGGCAAATGG
Lrg1	Forward primer	CTGGCACCAAGCTAAGCACAATC
	Reverse primer	TGGCAGTCTGTCTGGAAGTAAGC
Angptl4	Forward primer	GGC AGG AAC G AGTTTAG G C AAATC
	Reverse primer	AGGGAGAGAGTGAAGCAGGAGAG
GAPDH	Forward primer	CCGCCTGGAGAAACCTGTATGTATG
	Reverse primer	ATGCCTGCTTCACCACCTTCTTG

### Quantification and statistical analysis

All data were shown as mean ± SEM unless otherwise specified. Statistical analysis was performed with Origin 2021 software (OriginLab Corporation, USA) and SPSS Statistics 20 (IBM, USA). Behavioral experiments were recorded and analyzed with SMART software (USA). All data were tested for homogeneity of variance and normal distribution (Kolmogorov–Smirnov and Shapiro–Wilk tests) before the parametric hypothesis tests. The independent sample *t*-test was used, for example, in the Y-maze test for comparison. The significance levels were indicated as *p < 0.05, **p < 0.01, ***p < 0.001.

## Results

### Spatial cognition ability is enhanced in rats exposed to long-term SM

**FIGURE 1:**
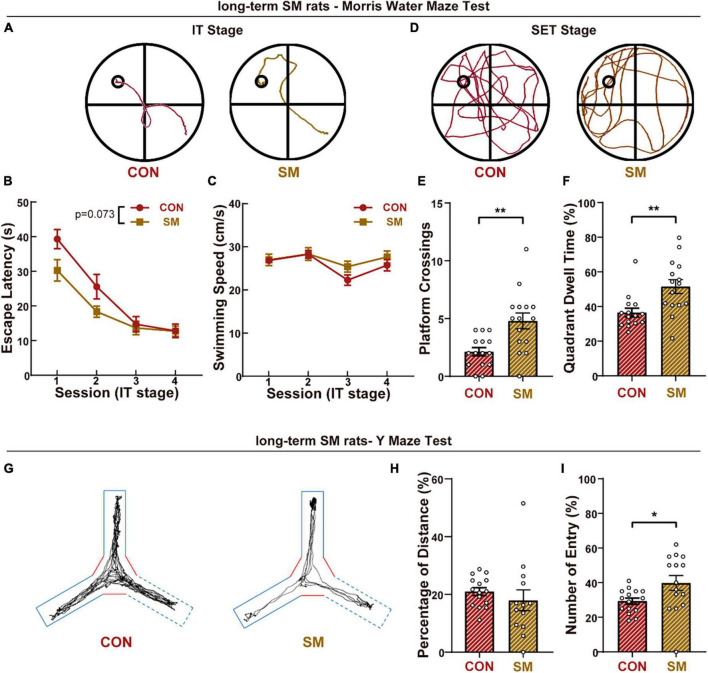
The performance of long-term SM exposure rats in working memory and advanced cognitive abilities. **(A)** Representative swim traces of two groups rats in the IT stage (CON: *n* = 15 rats, SM: *n* = 15 rats). **(B)** Mean escape latency for each session in the initial training stage. **(C)** Mean swimming speed for each session in the initial training stage. **(D)** Representative swim traces of two groups of rats in the SET stage. **(E)** Mean numbers of platform area crossings in RET stage. **(F)** Quadrant dwell time in space exploring test stage. **(G)** Representative traces of two groups rats in the Y-maze test. **(H)** Mean percentage of distance traveled in the novel arm of Y-maze. **(I)** Mean percentage of the entry into the novel arm of Y-maze. CON vs. SM, *p < 0.05; **p < 0.01.

We first explored the advanced cognitive performance under long-term SM exposure in rats and the long-term memory was examined by the MWM test. During the IT stage (Figure 1A), all rats were able to successfully learn the rule of this task, as shown by progressively shorter trajectories over the four sessions of the training stage and finished the session well (Figure 1B). Although it was suspected that the effect on skeletal muscles induced by SM might impact the swimming exploration in the MWM test, the comparison in swimming speed excluded the suspicion (Figure 1C). To further test the memory ability, rats’ traces in space exploration tests were recorded and analyzed (Figure 1D). Unexpectedly, compared to the CON group, the rats in SM group spent more time (Figure 1E, independent-sample *t*-test, *t*_(28)_ = −3.177, *p* = 0.004) and platform crossing (Figure 1F, independent-sample *t*-test, *t*_(28)_ = −3.441, *p* = 0.002) to explore the probe quadrant, suggesting the robust spatial memory.

Meanwhile, the short-term memory was evaluated by the Y-maze (Figure 1G). As reported before, the mean speed of rats exposed SM procedure was less than that of CON rats (Supplementary Figure 1A, independent-sample *t*-test, *t*_(28)_ = 2.364, *p* = 0.025), due to the effects of SM on motor performance. Then we measured the percentage of distance traveled in the novel arm, and no difference was found between the control group and SM group (Figure 1H). However, during the test of the Y-maze, we found that some rats would not like to explore the experimental space (Supplementary Figure 1B, independent-sample *t*-test, *t*_(28)_ = 6.373, *p* < 0.001). Thus we analyzed the percentage of entry traveled into the novel arm, and the SM rats entered the novel arm more (Figure 1I, independent-sample *t*-test, *t*_(28)_ = −2.276, *p* = 0.031). In addition to the gait disorder, the less exploration of SM rats might be also due to mood disorders. We then performed the open field test and EPM test to quantify the anxious condition of animals. Compared with the CON rats, SM rats exhibited less entry into the central zone in OPT (Supplementary Figure 1C, independent-sample *t*-test, *t*_(28)_ = 2.306, *p* = 0.029). Similarly, SM rats also traveled less distance in the open arms of the EPM test (Supplementary Figure 1D, independent-sample *t*-test, *t*_(28)_ = 2.286, *p* = 0.030). The data reflected that the SM model caused the anxious feeling in rats, consistent with our previous study. These results suggested that long-term SM exposure improved the cognition ability of rats.

### Synaptic functional plasticity of the hippocampal CA3-CA1 pathway is impaired in rats exposed to long-term SM

**FIGURE 2:**
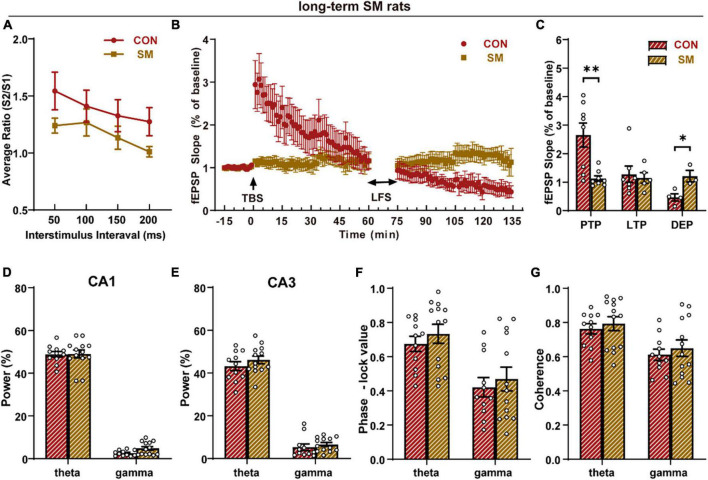
Synaptic plasticity and neural oscillation analysis of the hippocampus in rats exposed to long-term SM (CON: *n* = 11 rats, SM: *n* = 13 rats). **(A)** Paired-pulse ratios (PPR) of field excitatory postsynaptic potentials (fEPSPs). **(B)** The time coursing of normalized fEPSPs slopes recorded from CA3 to CA1 regions. **(C)** Histogram shows the average changes in fEPSPs slopes in PTP, LTP, and DEP. **(D)** The power spectral density of theta and gamma rhythms in the CA1 region. **(E)** The power spectral density of theta and gamma rhythms in the CA3 region. **(F)** Phase-locking value between hippocampal CA3 and CA1 regions in theta and gamma rhythms. **(G)** Coherence between hippocampal CA3 and CA1 regions in theta and gamma rhythms. CON vs. SM, *p < 0.05; **p < 0.01.

To investigate the underlying mechanisms of enhanced spatial memory function, we performed electrophysiological recording of the hippocampal CA3-CA1 pathway in rats to explore the alteration induced by SM. The synaptic plasticity of the hippocampus was determined by recording evoked field excitatory postsynaptic potentials (fEPSPs) in CA1 pyramidal neurons in response to CA3 stimulation. After the TBS, the response in the CA1 region became larger than the baseline (Figure 2B). The average short-term response (first 10 min) after TBS, often called post-tetanic potentiation (PTP), was reduced by SM procedure (Figure 2C, independent-sample *t*-test, *t*_(14)_ = 3.528, *p* = 0.003). However, the comparison of the average long-term response (LTP, last 10 min) after TBS showed that there was the same level among the three groups (Figure 2C). Following the recording of long-term potentiation, low-frequency stimulation (LFS) was performed to induce depotentiation (Figure 2B). In the first 10 min after LFS, there was no significant difference in the response between the CON rats and SM rats. Over time, however, the fEPSPs of CON rats became smaller and smaller. The average of fEPSPs in the last 10 min after LFS showed that the response of CON rats was less than SM rats (Figure 2C, independent-sample *t*-test, *t*_(5)_ = −3.275, *p* = 0.022). These results suggested that SM exposure impaired the synaptic plasticity of the hippocampal CA3-CA1 pathway in rats. Then, how about the presynaptic mechanism? To examine it, the paired-pulse ratio (PPR) was calculated, which was indeed affected mainly by presynaptic mechanisms. The results showed that there was the same ratio of the slope of the second response to that of the first between the two groups (Figure 2A), suggesting that the SM procedure did not affect the presynaptic function of the CA3-CA1 synapse. The above data suggested that long-term SM exposure impaired the synaptic plasticity of the hippocampal CA3-CA1 pathway.

Except for the hippocampal synapse function, neural oscillations of the hippocampus were also involved in the cognitive function. Thus, we analyzed the LFPs of hippocampal CA1 and CA3 regions. No significant difference was observed in the power spectral density of hippocampal CA1 (Figure 2D) and CA3 (Figure 2E) regions. Then we evaluated the phase synchronization and amplitude synchronization respectively and there was no significant difference in either theta rhythm or gamma rhythms, closely related to cognition function (Figures 2F, G). Thus, SM exposure did not impact the neural oscillation in hippocampus, which might support the intact spatial cognition under the damaged synaptic plasticity.

### The working memory performance of mice exposed to short- and long-term SM is enhanced

**FIGURE 3:**
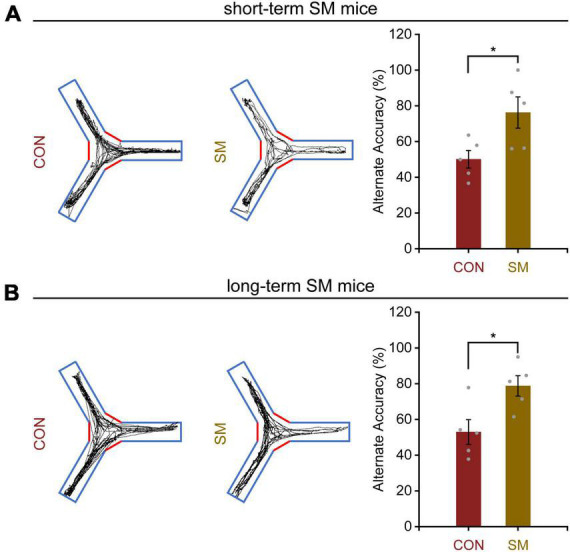
Working memory performance in mice exposed to short- and long-term SM. **(A)** In the Y-maze test, the position tracking from representative mice exposed to short-term SM and the alternate accuracy in 5 min, respectively (CON: *n* = 5 mice, SM: *n* = 5 mice). **(B)** In the Y-maze test, the position tracking from representative mice exposed to long-term SM and the alternate accuracy in 5 min, respectively. CON vs. SM, *p < 0.05.

To thoroughly investigate the disparities in behavior and synaptic plasticity alterations following exposure to SM, we opted to employ mice as subjects to unravel their deeper mechanisms at the genetic level and ascertain whether their intrinsic effects display time-dependent adaptive changes. Based on this, we first observed the working memory performance of mice subjected to short-term and long-term SM. It was found that mice exposed to short-term SM significantly increased their spontaneous alternation accuracy in the Y-maze (Figure 3A, independent-sample *t*-test, *t*_(8)_ = −2.610, *p* = 0.031), indicating that their working memory performance was better than that of normal mice. Surprisingly, as time went on, the long-term SM mice showed the same level of working memory capability as described above (Figure 3B, independent-sample *t*-test, *t*_(8)_ = −2.873, *p* = 0.021). These findings suggested that the working memory performance of mice was enhanced to some extent regardless of short- or long-term SM.

### Identification and comparison of DEGs from the RNA-seq of the hippocampus of mice exposed to short- and long-term SM

**FIGURE 4:**
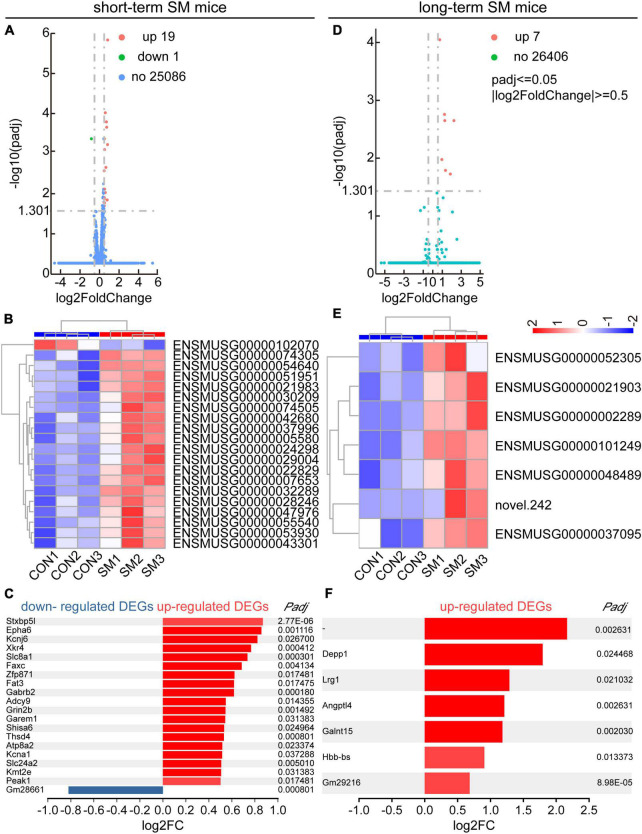
RNA-seq and DEGs analyses in the hippocampus of mice exposed to short- and long-term SM. **(A,D)** Volcano plot for the HPC RNA-seq analysis of mice exposed to short- and long-term SM, respectively (short-term SM: CON: *n* = 6 mice, SM: *n* = 6 mice; long-term SM: CON: *n* = 6 mice, SM: *n* = 6 mice). Differentially expressed genes (DEGs), defined by padj ≤ 0.05, | log2FC| ≥0.5. FC, foldchange. The complete lists of the RNA-seq analysis and DEGs are provided in Supplementary Tables 1, 2. **(B,E)** Hierarchical cluster analysis of DEGs among samples in the CON and SM groups exposed to short- and long-term SM, respectively (red, up-regulated; blue, down-regulated). **(C,F)** List of all upregulated and downregulated DEGs from the HPC RNA-seq analysis of mice exposed to short- and long-term SM, respectively.

**FIGURE 5:**
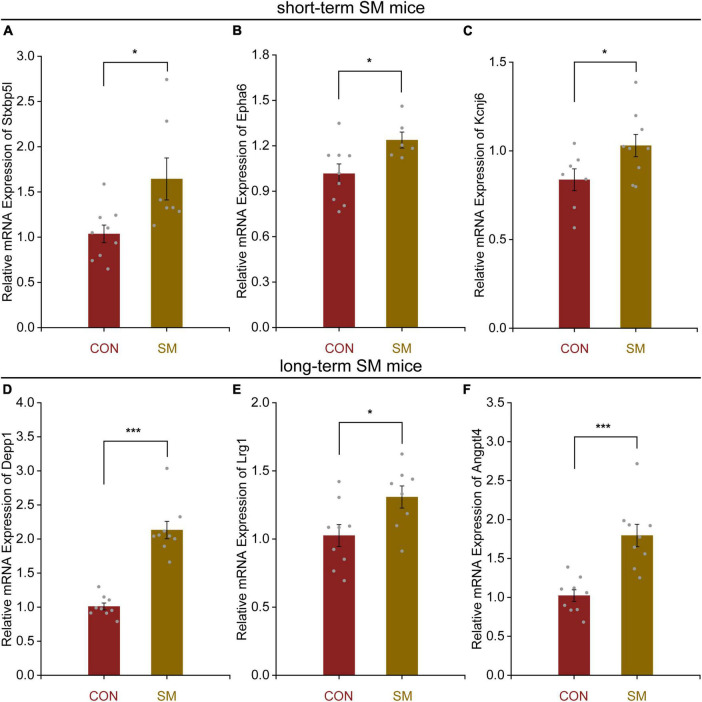
Quantitative real-time PCR verification for the top three DEGs in the hippocampus of mice exposed to short- and long-term SM in Figure 4. **(A–C)** Relative expression levels of genes *Stxbp5l*, *Epha6*, and *Kcnj6* in the hippocampus of mice after short-term SM (short-term SM: CON: *n* = 6 mice, SM: *n* = 6 mice). **(D–F)** Relative expression levels of genes *Depp1*, *Lrg1*, and *Angptl4* in the hippocampus of mice after long-term exposure to SM (long-term SM: CON: *n* = 6 mice, SM: *n* = 6 mice). ON vs. SM, *p < 0.05; ***p < 0.001.

Following this, we managed to obtain high quality RNA for RNA-seq analysis. In both the hippocampal regions of short- and long-term SM, basically, RNA-seq produced over 40.0 million raw reads from each sample. The percentage of clean reads reached more than 90.0%. Over 95% of the clean reads were mapped to the mouse genome (Supplementary Tables 1, 2). We reasoned that RNA-seq analysis, an unbiased approach, might highlight major molecular changes or signaling pathways in the hippocampus affected by SM exposure at different periods. After using the adjusted *p*-values (<0.05) for transcriptome analysis, we found that a total of 20 DEGs were identified in the hippocampus of mice exposed to short-term SM compared with the normal group, of which 19 were up-regulated and 1 was down-regulated (Figures 4A, B). Based on the fold change values, syntaxin binding protein 5-like (*Stxbp5l*), Eph receptor A6 (*Epha6*), and potassium inwardly rectifying channel, subfamily J, member 6 (*Kcnj6*) were the top three up-regulated genes (Figure 4C). We further verified the mRNA level by using RT-qPCR technology (Figures 5A–C, independent-sample *t*-test, Figure 5A: *t*_(8.118)_ = −2.412, *p* = 0.042; Figure 5B: *t*_(13)_ = −2.469, *p* = 0.028; Figure 5C: *t*_(14)_ = −2.146, *p* = 0.050). With the increase of modeling time, the DEGs became less and the gene types also changed. A total of seven DEGs were identified in mice exposed to long-term SM, all of which were up-regulated (Figures 4D, E). DEPP1 autophagy regulator (*Depp1*), leucine-rich alpha-2-glycoprotein 1 (*Lrg1*), and angiopoietin-like 4 (*Angptl4*) were the top three up-regulated genes (Figure 4F). The above DEGs expression was also confirmed at the mRNA level (Figures 5D–F, independent-sample *t*-test, Figure 5D: *t*_(16)_ = −8.172, *p* < 0.000; Figure 5E: *t*_(15)_ = −2.469, *p* = 0.026; Figure 5F: *t*_(16)_ = −4.776, *p* < 0.000). Moreover, the mean of log2 fold change of about the top 85% of DEGs after long-term SM exposure was higher than those after short-term SM, which indicated that the differences of DEGs after long-term SM exposure were more significant than that after short-term SM.

### Function-related families and signaling pathways of the hippocampus of mice exposed to short- and long-term SM

**FIGURE 6:**
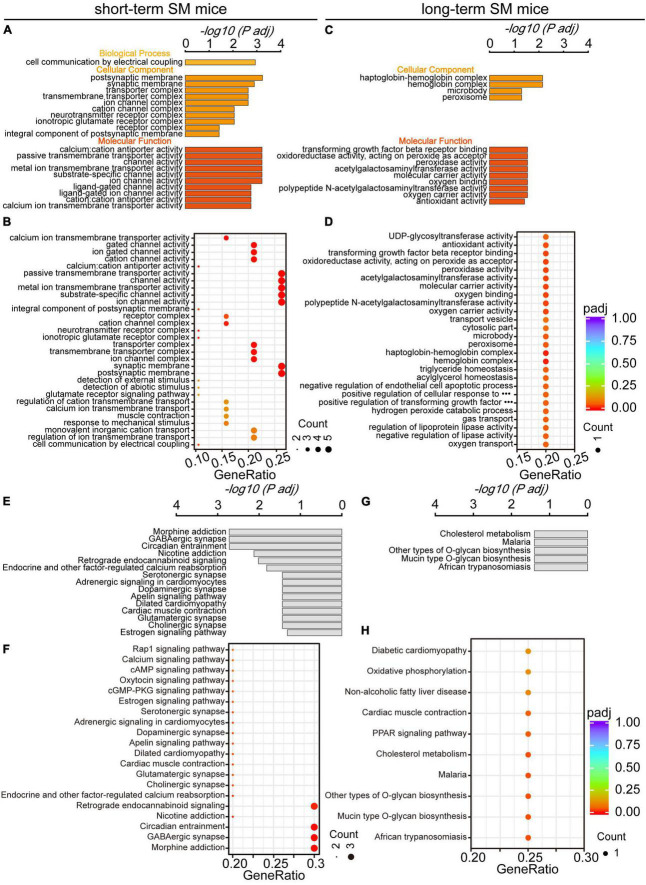
Gene Ontology and KEGG pathway analyses of HPC DEGs in mice exposed to short- and long-term SM, respectively. **(A,B)** All significantly enriched GO categories of biological process, cellular component, and molecular function terms between CON and SM groups exposed to short-term SM (short-term SM: CON: *n* = 6 mice, SM: *n* = 6 mice; long-term SM: CON: *n* = 6 mice, SM: *n* = 6 mice). **(C,D)** All significantly enriched GO categories of biological process, cellular component, and molecular function terms between CON and SM groups exposed to long-term SM. **(E,F)** All significantly enriched KEGG signaling pathways between CON and SM groups exposed to short-term SM. **(G,H)** All significantly enriched KEGG signaling pathways between CON and SM groups exposed to long-term SM.

Using GO analysis, we explored the effect of function-related biomolecular families in the hippocampus over SM time. There were obvious differences in the functional families of the hippocampus between short- and long-term SM exposure. After exposure to short-term SM, the enriched biological process was cell communication by electrical coupling. The enriched cellular components were the postsynaptic membrane, synaptic membrane, transporter complex, and so on. The enriched molecular functions were calcium: cation antiporter activity, passive transmembrane transporter activity, channel activity, and so on (Figures 6A, B). After exposure to long-term SM, the enriched cellular components were haptoglobin-hemoglobin complex, hemoglobin complex, microbody, and peroxisome. The enriched molecular functions were transforming growth factor beta receptor binding, oxidoreductase activity, acting on peroxide as an acceptor, peroxidase activity, and so on (Figures 6C, D).

In addition, we examined the alteration of signaling pathways in the hippocampus of mice exposed to SM at different periods using KEGG analysis. There were significant differences in hippocampal signaling changes between short- and long-term SM exposure. After exposure to short-term SM, morphine addiction, GABAergic synapse, circadian entrainment and nicotine addiction were significantly enriched (Figures 6E, F). After exposure to long-term SM, cholesterol metabolism, malaria, and other types of O-glycan biosynthesis were highly enriched (Figures 6G, H).

## Discussion

In the context of space missions, the space environment is a unique stressor that may impact the cognition function of astronauts. The level of cognitive ability plays a crucial role in determining the effectiveness of astronauts to complete their missions. However, current research on the effect of the space environment on cognitive function has not yielded conclusive results (Manzey and Lorenz, 1998; Qiong et al., 2016; Wollseiffen et al., 2016). The effects of microgravity exposure on cognition are likely to depend on the duration of exposure, and they can vary based on susceptibility, rate of recovery, and degree of adaptation (Ranjan et al., 2014).

Our behavioral results showed that both short- and long-term SM could enhance the working memory and complex cognitive ability of animals to the same extent. Similar results emerged in the previous study (Wollseiffen et al., 2016). It may be that lower levels of stress could benefit memory at encoding (Schwabe et al., 2012; Shields et al., 2017), and another example of this benefit of acute stress is students studying for exams. Experiencing mild to moderate stress during this learning phase could help them perform better on subsequent tests (Vogel and Schwabe, 2016). Showing that in the realm of cognitive function, both short- and long-term SM might be present as a less intense form of stress. In addition, as indicated in the results, the SM procedure indeed induced anxious emotion. These outcomes were in line with our earlier findings (Liang et al., 2022).

And space biologists worldwide are increasingly relying on omics approaches to maximize the knowledge gained from rare spaceflight experiments (Gertz et al., 2020; Rutter et al., 2020). Therefore, to investigate the reasons behind the changes in the for mentioned cognitive behaviors, we conducted transcriptome sequencing to analyze gene expression in the hippocampal regions exposed to short- and long-term SM, aiming to identify patterns during these periods and ascertaining whether their intrinsic effects display time-dependent adaptive changes. We opted to employ mice as subjects, and found that under short-term SM exposure, a total of 20 DEGs were identified in the hippocampus, with up-regulated genes accounting for 95% of the total DEGs. The top three upregulated DEGs were *Stxbp5l*, *Epha6*, and *Kcnj6*. Stxbp5l is known to play a role in regulating synaptic vesicular exocytosis and is involved in glucose homeostasis and secretion regulation within cells (Li et al., 2014). Epha6, which is involved in axon guidance, plays a crucial role in neuronal projection (Matsunaga et al., 2000; Petschner et al., 2013). Studies have revealed that mice exhibit impairments in learning and memory when the Epha6 protein is knocked out (Savelieva et al., 2008; Farini et al., 2020). Kcnj6, an essential component of the presynaptic membrane, serves as a key regulatory target for hippocampal neuron excitability and synaptic plasticity (Alfaro-Ruiz et al., 2021; Thompson and Satin, 2021; Chen et al., 2022). These up-regulated genes contribute to the regulation of synapse structure and function. GO and KEGG analyses further validate the aforementioned findings. It is hypothesized that this may explain why short-term SM exposure enhanced working memory abilities to some extent. Exploring the impact on behavior can be further investigated by manipulating the knockout or overexpression of relevant proteins, which is an area of great interest to us and will be our focus in future research.

Based on previous observations, it has been understood that the severity of the human body’s response to microgravity is somewhat dependent on the length of time an astronaut spends in space. Our findings aligned with this notion. We discovered that the mean of log2 fold change for approximately the top 85% of DEGs was higher under long-term SM exposure compared to short-term SM, indicating that the differences in DEGs were more significant following long-term SM exposure. Surprisingly, the number of DEGs was lower in individuals exposed to long-term SM, and all of them were upregulated. The top three upregulated DEGs were *DEPP1*, *Lrg1*, and *Angptl4*. DEPP1, which is located in mitochondria, is involved in the regulation of autophagy (Kuroda et al., 2010). It has been demonstrated that the DEPP1 protein plays a crucial role in coordinating thermogenesis by regulating adipocyte programs and may offer a potential target for treating metabolic disorders (Li et al., 2018; Guo et al., 2022). Lrg1, is known for its role in regulating cell proliferation (O’Connor et al., 2021; Pang et al., 2021). Angptl4 is involved in the negative regulation of apoptosis (Li et al., 2023; Son et al., 2023). Consequently, these DEGs appear to be more enriched in processes related to oxidative stress and metabolism. Moreover, the results of GO and KEGG analyses further support these findings. This is consistent with the results of other studies (Wang et al., 2017a). The above results revealed that there were adaptive changes in hippocampal DEGs expression based on the duration of modeling, which was time-dependent. The above findings provide unique insights into the temporal changes in hippocampal gene expression under microgravity conditions.

It is widely known that the synapse plays a critical role in a variety of cognitive processes (Buffington et al., 2014; Middei et al., 2014; Bin Ibrahim et al., 2022). Whether it participates the enhancement of cognition in SM exposure? Our previous findings indicated that long-term SM did not affect the structural plasticity of hippocampal synapses, such as dendrite spine density (Liang et al., 2022). However, it remained unknown how SM impacts functional plasticity. To explore this, we utilized the rat as a classic model organism for investigating synaptic function plasticity, employing electrophysiological recordings. Given the improved spatial memory performance observed in SM rats, we determined whether there were corresponding improvements in hippocampal synaptic function. Somewhat surprisingly, the results showed that after the SM procedure, synaptic plasticity in the hippocampal CA3-CA1 synapse was damaged. In most studies, there was a positive correlation between performance in learning and memory and the alteration in LTP. The majority of animal models with decreased LTP showed worsened learning and memory, and vice versa (Saxe et al., 2006; Lee and Silva, 2009; Han et al., 2013). So why did it lead to the above contradictory outcomes? On the one hand, some studies have shown that an increase in oxidative stress can lead to a decrease in synaptic protein expression, resulting in reduced intercellular signal transmission (Cai et al., 2016; Skaper et al., 2017; Thangwong et al., 2022). In our findings, prolonged exposure to SM could potentially induce excessive oxidative stress and metabolism, as mentioned in the RNA sequencing results above. It might cause a decline in synaptic function and potentially account for the observed decrease in hippocampal synaptic plasticity. However, it is crucial to note that this is merely a hypothesis and further comprehensive investigations are necessary. On the other hand, as a special stress, microgravity exposure causes structural and functional changes in the whole brain, resulting the complex and particular neuromechanism. This remains to be determined further.

## Conclusion

In summary, this study provides further insights into how cognitive function changes under SM exposure, which is consistently maintained or potentially even enhanced over both short and long durations. The underlying mechanisms are intricate and potentially linked to the contrasting expression of hippocampal-associated genes and alterations in synaptic function, with these effects being time-dependent. These findings offer fresh insights into the molecular mechanisms that underlie alterations in cognitive function in microgravity. As we venture toward space travel at an accelerated pace (Mars, Moon missions, etc.), the well-being of space crews becomes a paramount concern, and our discoveries can provide valuable guidance for exploring countermeasures and designing effective protective strategies in microgravity settings.

## Data availability statement

The original contributions presented in the study are publicly available. This data can be found here: https://zenodo.org/record/8383325.

## Ethics statement

The animal study was approved by the ARRIVE guidelines and should be carried out in accordance with the U.K. Animals (Scientific Procedures) Act, 1986 and associated guidelines, EU Directive 2010/63/EU for animal experiments, or the National Research Council’s Guide for the Care and Use of Laboratory Animals. The study was conducted in accordance with the local legislation and institutional requirements.

## Author contributions

RL: Data curation, Methodology, Software, Writing—original draft, Writing—review and editing. LW: Data curation, Funding acquisition, Writing—original draft, Writing—review and editing. QY: Conceptualization, Software, Writing—review and editing. QX: Investigation, Methodology, Writing—review and editing. SS: Data curation, Methodology, Writing—review and editing. HZ: Methodology, Supervision, Writing—review and editing. MZ: Investigation, Methodology, Writing—review and editing. JG: Investigation, Methodology, Writing—review and editing. CZ: Funding acquisition, Project administration, Resources, Writing—review and editing. JY: Funding acquisition, Project administration, Resources, Validation, Visualization, Writing—review and editing. DM: Funding acquisition, Project administration, Resources, Validation, Writing—review and editing.
