# Supplementary material for: Time-course adaptive changes in hippocampal transcriptome and synaptic function induced by simulated microgravity associated with cognition
Source: Front Cell Neurosci. 2023 Oct 5;17:1275771. doi: 10.3389/fncel.2023.1275771 (PMC10585108; doi:10.3389/fncel.2023.1275771)
Supplement: Supplementary file 1 [file Data_Sheet_1.PDF]

Fig. S1

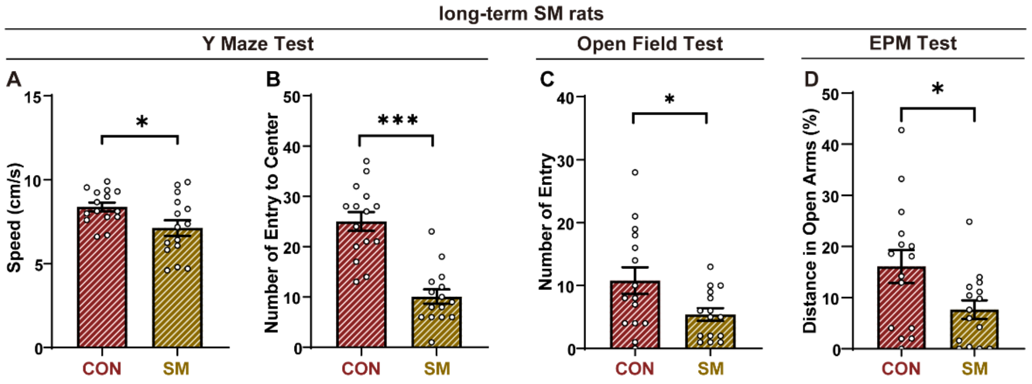

Fig. S1 The performance of long-term SM exposure rats in motor performance and emotional behaviors. A, B. Walking speed (**A**) and number of entries to center (**B**) of two groups rats in the Y-maze test. **C**. Number of entries to center of two groups rats in the open field test (CON: n=15 rats, SM: n=15 rats). **D.** Percentage of distance in open arms in elevated plus maze test (CON: n=15 rats, SM: n=15 rats).

Table. S1

Table S1 Evaluation and statistics of RNA sequencing valid data after short-term SM.

Sample ID	Raw Reads	Clean Reads	Clean Ratio	Mapping Ratio
CON1	45785258	43052922	94.03%	95.89%
CON2	40603076	37702986	92.86%	96.11%
CON3	44516420	41286960	92.75%	95.96%
SM1	45233776	41894534	92.62%	96.35%
SM2	43292940	40278356	93.04%	96.08%
SM3	45277838	41437326	91.52%	96.15%

Table. S2

Table S2 Evaluation and statistics of RNA sequencing valid data after long-term SM.

Sample ID	Raw Reads	Clean Reads	Clean Ratio	Mapping Ratio
CON1	44375692	43008990	96.92%	96.43%
CON2	44642150	43145216	96.65%	96.42%
CON3	46225864	44233682	95.69%	96.69%
SM1	45100638	43342774	96.10%	96.43%
SM2	44840874	42897642	95.67%	96.45%
SM3	44482044	42308204	95.11%	96.59%
